# Importance of presenting the variability of the false discovery rate control

**DOI:** 10.1186/s12863-015-0259-z

**Published:** 2015-08-04

**Authors:** Yi-Ting Lin, Wen-Chung Lee

**Affiliations:** Research Center for Genes, Environment and Human Health and Institute of Epidemiology and Preventive Medicine, College of Public Health, National Taiwan University, Rm. 536, No. 17, Xuzhou Rd., Taipei, 100 Taiwan

**Keywords:** Multiple testing, False discovery rate, Bootstrap

## Abstract

**Background:**

Multiple hypothesis testing is a pervasive problem in genomic data analysis. The conventional Bonferroni method which controls the family-wise error rate is conservative and with low power. The current paradigm is to control the false discovery rate.

**Results:**

We characterize the variability of the false discovery rate indices (local false discovery rates, q-value and false discovery proportion) using the bootstrapped method. A colon cancer gene-expression data and a visual refractive errors genome-wide association study data are analyzed as demonstration. We found a high variability in false discovery rate controls for typical genomic studies.

**Conclusions:**

We advise researchers to present the bootstrapped standard errors alongside with the false discovery rate indices.

**Electronic supplementary material:**

The online version of this article (doi:10.1186/s12863-015-0259-z) contains supplementary material, which is available to authorized users.

## Background

DNA microarray technology allows researchers to perform genome-wide screening and monitoring of expression levels for hundreds and thousands of genes simultaneously. The problem of multiple hypothesis testing arises when one compares a large number of genes between different groups (e.g., between breast cancer patients and healthy controls) [1]. In this context, the conventional Bonferroni method which controls the family-wise error rate is conservative and with low power. The current paradigm is to control the false discovery rate (FDR, the expected proportion of false positives among the rejected hypotheses) [2]. From a practicing epidemiologist’s viewpoint, the procedure is simple: input the *P*-values for the genes into an FDR software, get the output of the corresponding q-values [3], and then declare a gene significant if its q-value is less than or equal to 0.05. This supposedly ensures the FDR to be controlled at 5 % level.

If there are a total of *r* genes found to be significant using the above procedure, most researchers will reckon that the false positive genes among them would be no more than 0.05 × *r*. An interpretation such as these can be perilous. In fact, there are three levels of variations attached to any FDR control. The first level is the variation between the ‘local FDRs’. A local FDR for a gene is the probability of being false positive specifically for that gene [4–7]. The average local FDR of the *r* significant genes being 0.05 does not imply that all of them have a local FDR of 0.05. The second level of variation comes from the random errors in the estimation of the q-values themselves, which in turn relies on the empirical distribution function of the *P*-values. The fewer the genes are, the less stable the empirical distribution function is, and the more variable the estimated q-values will be. Finally, the total number of false positives by itself is a random variable. Its expected value being 0.05 × *r* does not guarantee that the actual number should be it.

In this paper, we use bootstrap method to characterize the variability of FDR control. A colon cancer gene-expression data [8] and a visual refractive errors genome-wide association study data [9] will be analyzed for demonstrations.

## Methods

Assume that there are a total *m* genes under study with *P*-values of *p*_*i*_, *i* = 1,…,*m*. From these, we calculate the local FDRs [4–7] and the q-values [3]: fdr_*i*_ and *q*_*i*_, for *i* = 1,…,*m*, respectively, using false discovery rate analysis package in R, such as fdrtool (specifying statistic = “*p*value”, plot = FALSE). Assume that among them there are a total of *r* (*r* > 0) genes with q-values at most as large as 0.05. We declare those genes significant with FDR controlled at 5 % level, and put them in an S set: S = {*i* : *q*_*i*_ ≤ 0.05}.

As the unit of analysis for an FDR control is a *P*-value rather than a study subject, we propose a *P*-value-based bootstrap method to characterize the variability of FDR control. Whereas the usual bootstrap method samples with replacement of the study subjects, our *P*-value-based bootstrap method samples with replacement directly of the *P*-values. This is computationally much more efficient, because the *P*-values in our method do not need to be re-computed from scratch for each bootstrapped sample as in the usual study-subject-based bootstrapping.

To be precise, the *j* th gene of a bootstrapped sample is *G*_*j*_ = [*m* × *U* + 1], where *U* is the uniform(0,1) distribution and [*x*] returns the largest integer not exceeding *x*. It has a *P*-value of ${p}_j^{*}={p}_{G_j}.$ From this new set of *P*-values: *p*_j_^*^ for *j* = 1,…,*m*., we calculate a new set of local FDRs: fdr_j_^*^ for *j* = 1,…,*m*. Note a star is superscripted to avoid confusion.

There is no guarantee that each and every gene in the original data will be represented in the bootstrapped sample. Put those ‘missing’ genes in a set: M = {*i* : *i* ≠ *G*_*j*_ for *j* = 1, …, *m*}. For an *i* ∉ M, we simply let its bootstrapped local FDR (superscripted B) be fdr_*i* ∉ M_^B^ = fdr_j_^*^, where *j* is any value satisfying *G*_*j*_=*i*. For an *i* ∈ M, we use linear interpolation to estimate its bootstrapped local FDR. First, we find its left and right ‘flanking’ genes. The left flanking genes are those that have the largest *P*-value (but no larger than *p*_*i*_) in the bootstrapped sample, that is, the set: $\mathrm{L}=\left\{j:{p}_j^{*}=\underset{p_k^{*}\le {p}_i}{ \max}\left({p}_k^{*}\right)\right\}$. The right flanking genes are those that have the smallest *P*-value (but no smaller than *p*_*i*_) in the bootstrapped sample, that is, the set: $\mathrm{R}=\left\{j:{p}_j^{*}=\underset{p_k^{*}\ge {p}_i}{ \min}\left({p}_k^{*}\right)\right\}$. If L is non-empty, we randomly pick one member in it, say *u*, and let *p*_*L*_ = *p*_u_^*^ and fdr_*L*_ = fdr_u_^*^. If L is empty, we let *p*_*L*_ = fdr_*L*_ = 0. If R is non-empty, we randomly pick one member in it, say *v*, and let *p*_*R*_ = *p*_v_^*^ and fdr_*R*_ = fdr_v_^*^. If R is empty, we let *p*_*L*_ = fdr_*L*_ = 1. Now we can use the linear interpolation. If *p*_*L*_ ≠ *p*_*R*_, the bootstrapped local FDR for this *i* ∈ M is ${\mathrm{fd}\mathrm{r}}_{i\in \mathrm{M}}^{\mathrm{B}}=\frac{\mathrm{fd}{\mathrm{r}}_R\times \left({p}_k-{p}_L\right)+\mathrm{f}\mathrm{d}{\mathrm{r}}_L\times \left({p}_R-{p}_k\right)}{p_R-{p}_L}$. If *p*_*L*_ = *p*_*R*_, we let fdr_*i* ∈ M_^B^ = fdr_*R*_ (fdr_*L*_ = fdr_*R*_ in this situation anyway).

In a bootstrapped sample, we calculate the bootstrapped q-value by simply averaging the bootstrapped local FDRs pertaining to the *r* significant genes, that is, ${q}^{\mathrm{B}}=\frac{1}{r}\times {\displaystyle \sum_{i\in \mathrm{S}}{\mathrm{fdr}}_i^{\mathrm{B}}}$. Next, we simulate a binary ‘false discovery indicator’ (1: false positive; 0: true positive) for each and every significant gene. The simulation is done according to an independent Bernoulli distribution with the corresponding bootstrapped local FDR as the parameter. The bootstrapped total number of false positives is then simply the summation of these false discovery indicators, and the bootstrapped false discovery proportion (FDP), that number divided by *r*, that is, ${\mathrm{FDP}}^{\mathrm{B}}=\frac{1}{r}\times {\displaystyle \sum_{i\in \mathrm{S}} Bernoulli\left({\mathrm{fdr}}_i^{\mathrm{B}}\right)}$. Note that of the *r* significant genes, the *q*^B^ is the average bootstrapped false discovery probability, and the FDP^B^, the bootstrapped proportion of false positives.

A total of 10,000 bootstrapped samples were generated to estimate the bootstrapped standard errors for the local FDRs, q-value and FDP, respectively. For independent genes, the 95 % bootstrapped percentile confidence intervals for local FDR and q-value at various *P*-value cutoffs can maintain the coverage probabilities close to the nominal value of 0.95, but for correlated genes, the coverage is below 0.95 (Additional file 1). In practice, it is difficult to tell whether the genes under study are independent of one another or are correlated. Therefore, the bootstrapped standard errors presented in this paper should better be regarded as lower bounds of the variability of the FDR control.

## Results

**Fig. 1:**
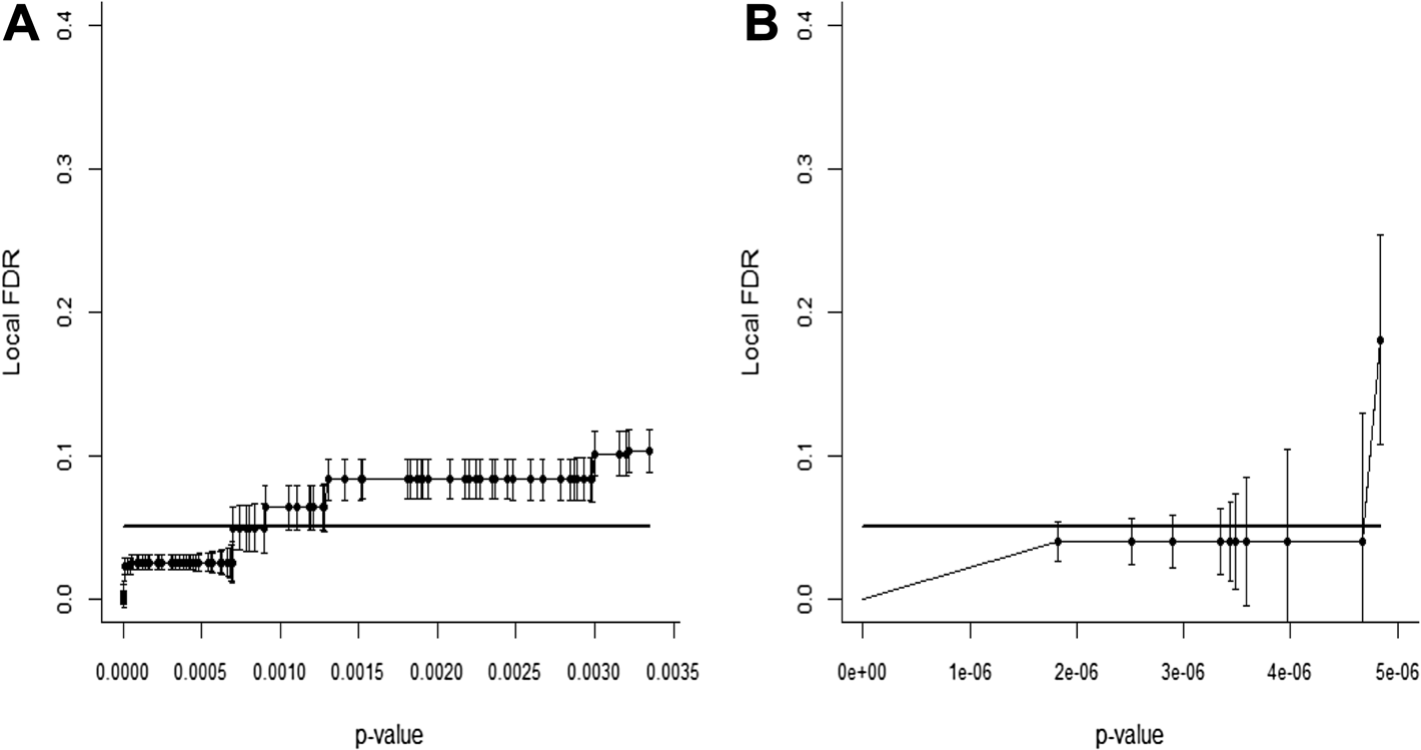
Local false discovery rates (FDRs) of significant genes in the colon cancer data (**a**) and the refractive errors data (**b**). Error bars are ± 1 bootstrapped standard error. The bold line marks the FDR control value of 0.05

The colon cancer data of Alon et al. [8] contains the gene expression measurements of 2000 genes for 62 samples including 40 colon cancer tissue samples and 22 normal tissue samples. The *P*-value of each gene is calculated by Student’s *t*-test. A total of 95 significant differentially expressed genes are found with FDR controlled at 5 % level. Figure 1a shows the local FDRs. We see that their local FDR values are not all controlled at 0.05. A total of 43 significant genes have local FDR values larger than 0.05, and the largest one is 0.10. Using the bootstrap method, we can gauge the variability of the FDR control. We see that the largest bootstrapped standard error for the local FDRs is 0.017 (Fig. 1a). The bootstrapped standard error for the q-value is 0.006, and for the FDP, an upward of 0.023 (Table 1).

**Table 1:** The bootstrapped standard errors of q-value and false discovery proportion (FDP) among significant genes

	Bootstrapped standard errors
Colon cancer data	
q-value	0.0060
FDP	0.0234
Refractive errors data	
q-value	0.0273
FDP	0.0828

The visual refractive errors data of Stambolian et al. [9] consists of genome-wide association studies for 7280 samples from five cohorts. We choose the data from chromosome 14 which is composed of 84,536 single nucleotide polymorphisms (SNPs). The *P*-value of each SNP is calculated from meta-analysis of five cohorts. There are ten significant SNPs detected with FDR controlled at 5 % level. Figure 1b shows the local FDRs. Although most of their local FDR values are near 0.05, the largest one is 0.18 which is a far cry from a FDR control of 5 %. Using the bootstrap method, we find the variability of the FDR control in this data to be even greater than that in the colon cancer data. For the local FDRs, the largest bootstrapped standard error can be as large as 0.089 (Fig. 1b). For q-value and FDP, their bootstrapped standard errors are up to 0.027 and 0.083, respectively (Table 1).

## Discussion

Previous researchers [10–12] studied the variability of FDR control using computer simulation and found a number of factors associated with high variability: small sample size, small total number of genes, large correlation among the genes, and low signal prevalence/strength for the genes, etc. These researchers investigated one factor at a time. In real practice however, we need to gauge the overall effect of multiple factors. In this study, we propose a simple bootstrap method to characterize the three levels of variations (local FDRs, q-value, and FDP) associated with an FDR control. A small-scale simulation in Additional file 2 shows that the results of the present method are in agreement with the previous computer simulation studies. However, the present method is completely data-driven, requiring no *a priori* knowledge about which factor(s) might influence the variability and by how much. Using a simple bootstrap procedure, the methods automatically takes into account all factors that may influence the variability of FDR control. Additional file 3 presents handy R codes for implementing the method.

In this study, we found the variability in FDR controls to be quite large for the colon cancer gene expression and the visual refractive errors genome-wide association study data. [The computer-simulation methods of Gold et al. [10], Green and Diggle [11], and Zhang and Coombes [12] cannot be directly applied to these datasets for comparisons, because their methods require extra information beyond the data at hand.] We also found a potential danger in using the q-value to infer significance. Take the visual refractive errors data as an example. Using the criterion of q ≤0.05, a total of ten significant SNPs can be detected. However, one of them actually has a local FDR as large as 0.18. Clearly, it is too liberal to declare a SNP with such high rate of false positive to be significant. If the significance of a particular gene is at issue, naturally we must turn to its local FDR (and the associated bootstrapped standard error), rather than its q-value. Only when a gene has a very low local FDR value, can it be pretty safe to declare that gene significant, for example, when its local FDR value plus two standard errors is still lower than 0.05.

## Conclusions

This study demonstrates the high variability in FDR controls for typical genomic studies. To avoid over-interpretations, researchers are advised to present the associated bootstrapped standard errors alongside with the FDR indices of local FDRs, q-value and FDP.

## Additional files

Additional file 1:
**A simulation study for coverage probabilities.** (DOC 46 kb) Additional file 2:
**A simulation study for standard errors.** (DOCX 20 kb) Additional file 3:
**R codes.** (DOC 30 kb)

## Abbreviations

FDR: False discovery rate
FDP: False discovery proportion
SNP: Single nucleotide polymorphism

## References

[1] Pounds SB (2006). Estimation and control of multiple testing error rates for microarray studies. Brief Bioinform.

[2] Benjamini Y, Hochberg Y (1995). Controlling the false discovery rate - a practical and powerful approach to multiple testing. J Roy Stat Soc (B).

[3] Storey JD, Tibshirani R (2003). Statistical significance for genomewide studies. Proc Natl Acad Sci U S A.

[4] Efron B (2004). Large-scale simultaneous hypothesis testing: the choice of a null hypothesis. J Am Stat Assoc.

[5] Liao JG, Lin Y, Selvanayagam ZE, Shih WJ (2004). A mixture model for estimating the local false discovery rate in DNA microarray analysis. Bioinformatics.

[6] Scheid S, Spang R (2004). A stochastic downhill search algorithm for estimating the local false discovery rate. IEEE/ACM Trans Comput Biol Bioinform.

[7] Strimmer K (2008). A unified approach to false discovery rate estimation. BMC Bioinform.

[8] Alon U, Barkai N, Notterman DA, Gish K, Ybarra S, Mack D (1999). Broad patterns of gene expression revealed by clustering analysis of tumor and normal colon tissues probed by oligonucleotide arrays. Proc Natl Acad Sci U S A.

[9] Stambolian D, Wojciechowski R, Oexle K, Pirastu M, Li X, Raffel LJ (2013). Meta-analysis of genome-wide association studies in five cohorts reveals common variants in RBFOX1, a regulator of tissue-specific splicing, associated with refractive error. Hum Mol Genet.

[10] Gold DL, Miecznikowski JC, Liu S (2009). Error control variability in pathway-based microarray analysis. Bioinformatics.

[11] Green GH, Diggle PJ. On the operational characteristics of the Benjamini and Hochberg false discovery rate procedure. Stat Appl Genet Mol Biol. 2007;6: Article27. 10.2202/1544-6115.1302 18052910

[12] Zhang J, Coombes KR (2012). Sources of variation in false discovery rate estimation include sample size, correlation, and inherent differences between groups. BMC Bioinform.

