# Supplementary material for: Importance of presenting the variability of the false discovery rate control
Source: BMC Genet. 2015 Aug 4;16:97. doi: 10.1186/s12863-015-0259-z (PMC4523994; doi:10.1186/s12863-015-0259-z)
Supplement: Additional file 1: — A simulation study for coverage probabilities. (DOC 46 kb) [file 12863_2015_259_MOESM1_ESM.doc]

**Additional file 1.** A simulation study for coverage probabilities.

First, we simulate a very large gene pool (a total of 10^8^ genes) to approximate the true values of the local FDR and q-value at different P-values. Test statistics for the noise genes (true negatives) follow the standard normal distribution, those for the signal genes (true positives) (with the prevalence of 0.4), the normal distribution with a standard deviation of 1.0 and a mean of 3.0 (strong signal strength) and 1.5 (weak signal strength), respectively. The genes are assumed to be independent to one another. Next, the simulated test statistics are converted to the P-values according to the complementary cumulative density function of the standard normal distribution (P-value$=1-\Phi(x)$ for a test statistic $x$). The total 10^8^ P-values allow us to very accurately approximate the P-value distribution function, from which the true values of the local FDRs and q-values at different P-value cutoffs can be calculated. In addition, we also perform simulations for correlated genes. We use block structure (with 50 genes in one block) to simulate dependency between genes. The covariance between any two genes within the same block is set to be 0.3 (moderate correlation) and 0.1 (weak correlation), whereas genes in different block are assumed to be independent to one another.

A total of 1000 simulations are then performed to study the coverage probability of the proposed bootstrapped method for each scenario. In each round of the simulation, for independent genes, we randomly sample 1000 genes from the gene pool of the 10^8^ genes as the data to be bootstrapped. For correlated genes, we randomly sample 20 blocks. In each of 1000 bootstrapped samples, the local FDRs and q-values at different P-value cut-off points are calculated. From these, the 95% bootstrapped percentile confidence intervals are calculated.

The coverage probabilities for the proposed bootstrapped method are shown below:

For independent genes:

| P-value cutoffs | Weak signal strength | | Strong signal strength | |
| --- | --- | --- | --- | --- |
|  | Local FDR | q-value | Local FDR | q-value |
| 0.001 | 0.9538 | 0.9457 | 0.9585 | 0.9480 |
| 0.003 | 0.9563 | 0.9545 | 0.9589 | 0.9462 |
| 0.005 | 0.9584 | 0.9499 | 0.9595 | 0.9516 |
| 0.010 | 0.9603 | 0.9601 | 0.9571 | 0.9531 |
| 0.030 | 0.9569 | 0.9607 | 0.9598 | 0.9487 |
| 0.050 | 0.9642 | 0.9657 | 0.9615 | 0.9472 |
| 0.100 | 0.9650 | 0.9638 | 0.9574 | 0.9466 |

For genes with weak correlation:

| P-value cutoffs | Weak signal strength | | Strong signal strength | |
| --- | --- | --- | --- | --- |
|  | Local FDR | q-value | Local FDR | q-value |
| 0.001 | 0.8934 | 0.8298 | 0.9581 | 0.8746 |
| 0.003 | 0.9014 | 0.7970 | 0.9611 | 0.8999 |
| 0.005 | 0.8957 | 0.7827 | 0.9676 | 0.9161 |
| 0.010 | 0.9005 | 0.7725 | 0.9621 | 0.9318 |
| 0.030 | 0.9028 | 0.7579 | 0.9592 | 0.9388 |
| 0.050 | 0.9088 | 0.7578 | 0.9503 | 0.9258 |
| 0.100 | 0.9084 | 0.7688 | 0.9193 | 0.9105 |

For genes with moderate correlation:

| P-value cutoffs | Weak signal strength | | Strong signal strength | |
| --- | --- | --- | --- | --- |
|  | Local FDR | q-value | Local FDR | q-value |
| 0.001 | 0.7742 | 0.6333 | 0.9430 | 0.6736 |
| 0.003 | 0.7647 | 0.5905 | 0.9591 | 0.7197 |
| 0.005 | 0.7604 | 0.5766 | 0.9604 | 0.7476 |
| 0.010 | 0.7657 | 0.5648 | 0.9627 | 0.7782 |
| 0.030 | 0.7789 | 0.5476 | 0.9460 | 0.8068 |
| 0.050 | 0.7798 | 0.5407 | 0.9061 | 0.7795 |
| 0.100 | 0.7969 | 0.5476 | 0.8182 | 0.7533 |
