# Supplementary material for: Importance of presenting the variability of the false discovery rate control
Source: BMC Genet. 2015 Aug 4;16:97. doi: 10.1186/s12863-015-0259-z (PMC4523994; doi:10.1186/s12863-015-0259-z)
Supplement: Additional file 2: — A simulation study for standard errors. (DOCX 20 kb) [file 12863_2015_259_MOESM2_ESM.docx]

**Additional file 2.** A simulation study for standard errors.

Test statistics are simulated first. Those for the noise genes (true negatives) follow the standard normal distribution, those for the signal genes (true positives), the normal distribution with a standard deviation of 1.0 and a mean of 3.0 (strong signal strength) and 1.5 (weak signal strength), respectively. The genes are assumed to be independent to one another. Next, the simulated test statistics are converted to the P-values according to the complementary cumulative density function of the standard normal distribution (P-value$=1-\Phi(x)$ for a test statistic $x$). The total number of genes is examined for large (5000) and small (1000), and the signal prevalence, for high (0.4) and low (0.1), respectively. In addition, we also perform simulations for correlated genes (only for the settings of small total number of genes: 1000). We use block structure to simulate dependency between genes. We divide the genes into 100 blocks (each with 10 genes). The covariance between any two genes within the same block is set to be 0.3, whereas genes in different block are assumed to be independent to one another.

A total of 1000 simulations are performed for each scenario. In each round of the simulations, a total of 100 bootstrapped samples are generated. We randomly pick a signal gene that achieves significance to calculate the average local FDR for the signal genes. For the calculations of the q-value and FDP, see text. The bootstrapped standard errors for these FDR indices are shown in the following table (shown in parentheses are the results for correlated genes):

|  | Bootstrapped standard errors | | |
| --- | --- | --- | --- |
|  | Average  local FDR  for signal genes | q-value | FDP |
|  |  |  |  |
| Large total number |  |  |  |
| Strong signal strength |  |  |  |
| High signal prevalence | 0.0116 | 0.0023 | 0.0053 |
| Low signal prevalence | 0.0275 | 0.0041 | 0.0123 |
| Weak signal strength |  |  |  |
| High signal prevalence | 0.0295 | 0.0038 | 0.0135 |
| Low signal prevalence | 0.0442 | 0.0238 | 0.1085 |
| Small total number |  |  |  |
| Strong signal strength |  |  |  |
| High signal prevalence | 0.0213  (0.0228) | 0.0055 (0.0063) | 0.0119  (0.0135) |
| Low signal prevalence | 0.0551  (0.0711) | 0.0126  (0.0502) | 0.0298  (0.0641) |
| Weak signal strength |  |  |  |
| High signal prevalence | 0.0530  (0.0520) | 0.0082  (0.0106) | 0.0311  (0.0526) |
| Low signal prevalence | 0.0772 | 0.0536 | 0.1833 |
|  | (0.0695) | (0.0650) | (0.1715) |
|  |  |  |  |
