# Supplementary material for: Importance of presenting the variability of the false discovery rate control
Source: BMC Genet. 2015 Aug 4;16:97. doi: 10.1186/s12863-015-0259-z (PMC4523994; doi:10.1186/s12863-015-0259-z)
Supplement: Additional file 3: — R codes. (DOC 30 kb) [file 12863_2015_259_MOESM3_ESM.doc]

**Additional file 3.** R codes.

# Use the colon cancer data as demonstration

# Import the P-value of the data

pvalue <- read.table("Colon cancer.txt")

# Estimate local FDRs of genes in the data

library(fdrtool)

locfdr <- fdrtool(pvalue,statistic=”pvalue”,plot=FALSE)

locfdr$lfdr

# Calculate local FDRs and interpolated local FDRs in bootstrapped samples

library(fdrtool)

sig <- 95

ntest <- 2000

Bt <- 10000

result <- matrix(nrow=sig,ncol=Bt)

for(i in 1:Bt){

boot <- sample(pvalue$p,ntest,replace=TRUE)

a <- fdrtool(boot,statistic="pvalue",plot=FALSE)

localfdr <- a$lfdr

b <- localfdr[order(localfdr,decreasing=FALSE)]

orderlfdr <- rep(NA,(ntest+2))

orderlfdr[1] <- 0

orderlfdr[2:(ntest+1)] <- b

orderlfdr[(ntest+2)] <- 1

c <- boot[order(boot,decreasing=FALSE)]

orderboot <- rep(NA, (ntest+2))

orderboot[1] <- 0

orderboot[2: (ntest+1)] <- c

orderboot[(ntest+2)] <- 1

for(k in 1:sig){

c <- which(orderboot== pvalue$p[k])

result[k,i] <- orderlfdr[c[1]]

}

d <- which(is.na(result[,i]))

for(w in 1:length(d)){

dd <- pvalue$p[d[w]]

left <- max(which(orderboot<dd))

lp <- orderboot[left]

llfdr <- orderlfdr[left]

right <- min(which(orderboot>dd))

rp <- orderboot[right]

rlfdr <- orderlfdr[right]

result[d[w],i] <- (rlfdr*dd-rlfdr*lp -llfdr*dd+llfdr*rp)/(rp-lp)

}

}

# Bootstrapped standard error of q-value

average <- rep(NA,Bt)

for(x in 1:Bt){

average[x] <- mean(result[1:sig,x])

}

sd(average)

# Bootstrapped standard error of local FDRs

sdvalue <- rep(NA,sig)

for(y in 1:sig){

sdvalue[y] <- sd(result[y,1:10000])

}

# Bootstrapped standard error of FDP

count <- matrix(nrow=sig,ncol=Bt)

for(bb in 1:Bt){

for(cc in 1:sig){

count[cc,bb] <- rbinom(1,1,result[cc,bb])

}

}

FDP <- rep(NA,Bt)

for(dd in 1:Bt){

hh <- which(count[,dd]==1)

FDP[dd] <- length(hh)/sig

}

sd(FDP)
